# Erdr1 Suppresses Murine Melanoma Growth via Regulation of Apoptosis

**DOI:** 10.3390/ijms17010107

**Published:** 2016-01-14

**Authors:** Joohyun Lee, Min Kyung Jung, Hyun Jeong Park, Kyung Eun Kim, Daeho Cho

**Affiliations:** 1 Department of Life Systems, Sookmyung Women’s University, Chungpa-Dong 2-Ka, Yongsan-ku, Seoul 140-742, Korea; joohyunlee214@gmail.com (J.L.); alsrud890@hanmail.net (M.K.J.); 2 Department of Dermatology, Yeouido St. Mary’s Hospital, The Catholic University of Korea, Seoul 150-713, Korea; hjpark@catholic.ac.kr

**Keywords:** Erdr1, melanoma, apoptosis, Bcl-2, Bax

## Abstract

Melanoma, one of the aggressive cancers, is known to be resistant to chemotherapy. Because of its aggressive nature, effectively inducing apoptosis is necessary to treat melanoma. Erythroid differentiation regulator 1 (Erdr1) is known to be a stress-related survival factor exhibiting anti-cancer effects in several cancers. However, little is known about the functions and underlying mechanisms of Erdr1 so far. To demonstrate the effect of Erdr1 in melanoma apoptosis, recombinant murine Erdr1 was injected into mice implanted with B16F10 melanoma cells. *In vivo* tumor growth was significantly inhibited in mice injected with Erdr1 compared to the control. In addition, the tumor from Erdr1-injected mice showed an increased level of apoptosis. Accordingly, apoptosis-regulating factors including anti-apoptotic marker Bcl-2 and pro-apoptotic marker Bax in the tumor tissues were examined. As expected, the decreased level of Bcl-2 and increased level of Bax were detected in tumors within the mice injected with Erdr1. Based on the *in vivo* study, the role of Erdr1 in tumor apoptosis was further tested by incubating it with cells of the murine melanoma cell line B16F10. Erdr1-induced apoptosis in B16F10 cells was observed. Additionally, Erdr1 downregulated STAT3 activity, inhibiting apoptosis via regulation of the Bcl-2 family. Overall, data demonstrate that Erdr1 induced murine melanoma apoptosis through the regulation of Bcl-2 and Bax. These findings suggest that Erdr1 is a novel regulator of apoptosis in melanoma.

## 1. Introduction

Melanoma is highly resistant to many conventional chemotherapeutic drugs due to aberrant regulation of the apoptosis signaling pathway [1]. During melanoma progression, various resistant mechanisms are formed, inhibiting the efficacy of currently available chemotherapeutic drugs. Although available treatment strategies have been developed in order to minimize resistance from occurring, they still have several obstacles such as cost and side effects [2]. Hence, understanding the precise mechanism of melanoma apoptosis and identification of effective apoptosis inducers are essential for the development of an effective therapy for this aggressive cancer.

Signal Transducer and Activator of Transcription 3 (STAT3) regulates various aspects of cancer cell biology, including apoptosis, proliferation, and survival. In addition, STAT3 represses apoptosis via regulation of Bcl-2 and its family members [3]. Specifically, STAT3 is hyperactivated in melanoma and contributes to chemoresistance [4].

Erdr1 has been known as a protein that can induce hemoglobin synthesis in both human and murine erythroleukemia [5]. Although only a few studies on Erdr1 have been reported, several have suggested that Erdr1 exerts anti-cancer effects in various cancers, including melanoma and gastric cancer [6,7]. Also, Erdr1 exhibits these functions not only in cancer cells but also in immune cells. Treatment of natural killer cells with recombinant Erdr1 enhances its cytotoxicity via the secretion of lytic granules [8], suggesting that this treatment increases the killing of cancer cells by these immune cells.

A representative proinflammatory cytokine, interleukin (IL)-18, exerts procancer effects in melanoma [9]. Erdr1, which is inversely correlated with IL-18, exhibits a negative role in melanoma progression [6]. In addition, the overexpression of Erdr1 increases ultraviolet B (UVB)-induced apoptosis in human keratinocytes [10], indicating that Erdr1 has a proapoptotic effect under these conditions. Based on these previous studies, we investigated the role of Erdr1 in melanoma apoptosis.

## 2. Results

### 2.1. Recombinant Murine Erdr1 Inhibits Tumor Growth in Vivo

**Figure 1:**
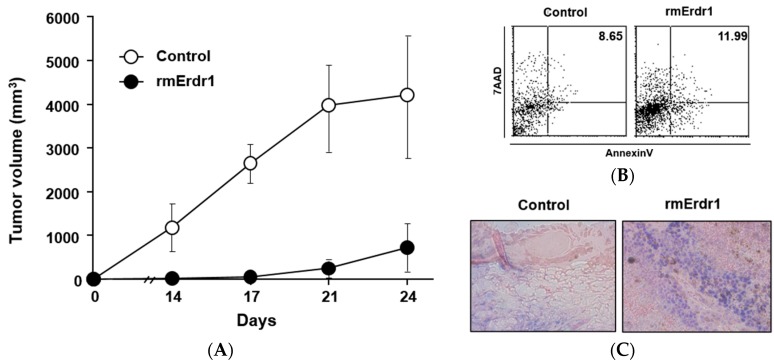
Erdr1 suppresses tumor growth as a result of increased apoptosis *in vivo*. (**A**) B16F10 cells were subcutaneously injected into six-week-old female C57BL/6 mice. Mice were then treated with PBS or Erdr1 (100 μg/kg) via intraperitoneal injection. Tumor size was measured with calipers every two days for 24 days; (**B**) Tumors were removed from C57BL/6 mice injected with PBS or Erdr1 (100 μg/kg). Four weeks after implantation of B16F10 cells, cells were isolated from tumor tissues and stained with Annexin V and 7-AAD to examine apoptosis. After staining, flow cytometry analysis was performed using FACS Calibur; (**C**) Tumors removed from the mice were cut into sections to detect *in situ* apoptosis using a terminal deoxynucleotidyl transferase dUTP nick end labeling (TUNEL) assay. Tissue sections were stained as mentioned in Materials and Methods. After staining, stained slides were examined by microscope and photographed. Original magnification (100×).

To determine the effect of Erdr1 on murine melanoma growth *in vivo*, vehicle control (phosphate-buffered saline (PBS)) or recombinant murine Erdr1 (rmErdr1) was injected intraperitoneally into C57BL/6 mice implanted with B16F10 melanoma cells. The tumor size of mice treated with recombinant murine Erdr1 was significantly smaller than that in mice treated with the vehicle (Figure 1A), suggesting that Erdr1 suppressed tumor growth. As tumor volume is determined by various processes including apoptosis and proliferation, apoptosis by recombinant murine Erdr1 treatment was measured. Four weeks after inoculation, cells were isolated from tumors and stained with 7-aminoactinomycin D (7-AAD) and Annexin V. Compared to PBS-treated mice, Erdr1-treated mice exhibited higher levels of apoptosis (Figure 1B). Increased apoptosis in tumor tissues following Erdr1 treatment was also confirmed using a TUNEL assay (Figure 1C). Taken together, these results suggest that Erdr1 induces apoptosis of melanoma cells *in vivo*, leading to decreased tumor volume.

### 2.2. Erdr1 Induces Apoptosis via Regulation of Bcl-2 and Bax in Vivo

**Figure 2:**
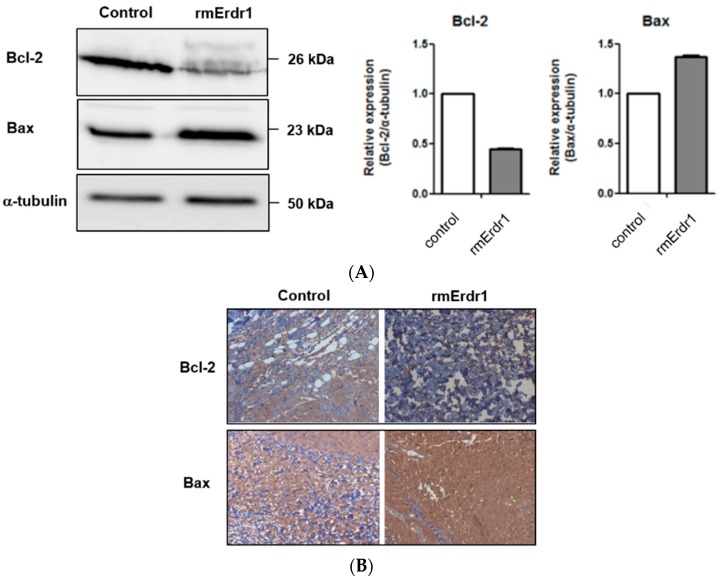
Erdr1 suppresses Bcl-2 and induces Bax *in vivo*. (**A**) Tumor tissues were lysed, and identical amounts of lysate were loaded onto an acrylamide gel. Western blotting was performed using rabbit anti-mouse Bcl-2 antibody, rabbit anti-mouse Bax antibody, and mouse anti-α-tubulin antibody; (**B**) Bcl-2 and Bax levels in tumor tissues were measured using immunohistochemistry. Sections from tumors were stained with rabbit anti-mouse Bcl-2 antibody and rabbit anti-mouse Bax antibody. Stained sections were examined with the microscope and photographed. Original magnification (100×).

As Bcl-2 family members are key regulators of apoptosis [3], the expressions of anti-apoptotic Bcl-2 and pro-apoptotic Bax were measured in tumor tissues of mice treated with either Erdr1 or the vehicle. Interestingly, Bcl-2 levels were significantly decreased whereas Bax levels were increased in tumors from Erdr1-treated mice compared to tumors from vehicle-treated mice (Figure 2A). Immunohistochemistry also confirmed the downregulation of Bcl-2 and upregulation of Bax following Erdr1 treatment (Figure 2B), suggesting that Erdr1 regulates apoptosis-regulating factors and results in increased apoptosis *in vivo*.

### 2.3. Erdr1 Induces Apoptosis in Murine Melanoma Cells, B16F10 in Vitro

**Figure 3:**
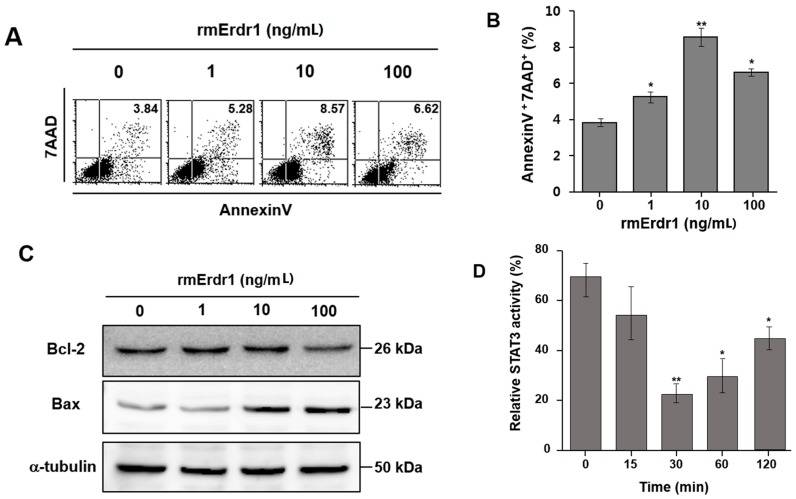
Erdr1 induces apoptosis via regulation of Bcl-2 and Bax in murine melanoma cell lines. (**A**) B16F10 cells were treated with different doses (0, 1, 10, and 100 ng/mL) of recombinant murine Erdr1 for 24 h. Staining with 7-AAD and Annexin V was performed to measure apoptosis induced by recombinant murine Erdr1. After staining, stained cells were analyzed with flow cytometry analysis. The image shows one of the representative experiments of three experiments performed independently; (**B**) Flow cytometry analysis data were converted into a bar graph. The data represents the mean ± SD of one of three independent experiments. *, *p* < 0.05 control *vs.* recombinant murine Erdr1 treatment groups. **, *p* < 0.01 control *vs.* recombinant murine Erdr1 treatment groups; (**C**) Bax and Bcl-2 were measured using Western blot. B16F10 cells were treated with different doses (0, 1, 10, and 100 ng/mL) of Erdr1 for 24 h. After lysis, equal amounts of protein lysates were loaded onto an acrylamide gel. To detect Bax and Bcl-2 expression, rabbit anti- mouse Bax antibody and rabbit anti- mouse Bcl-2 antibody were used; (**D**) The activity of STAT3 was measured in melanoma B16F10 cells treated with or without recombinant murine Erdr1 (10 ng/mL) after 0, 15, 30, 60, and 120 min. Cells were collected and lysed with lysis buffer. Subsequently, lysates were incubated with an oligonucleotide containing a STAT3 consensus binding site. After incubation, STAT3 activity was measured using an ELISA based on horseradish peroxidase (HRP) reaction. The data represents the mean ± SD of one of three independent experiments. *, *p* < 0.05 control *vs.* recombinant murine Erdr1 treatment groups. **, *p* < 0.01 control *vs.* recombinant murine Erdr1 treatment groups.

Additionally, recombinant murine Erdr1 was incubated with cells of the murine melanoma cell line B16F10, and then the cells were stained with 7-AAD and Annexin V to confirm the Erdr1-mediated apoptosis that was observed in melanoma *in vivo*. Apoptosis of B16F10 cells was significantly increased by treatment with recombinant murine Erdr1 compared to the control treatment (Figure 3A,B). To determine whether Erdr1 regulates Bax and Bcl-2 in B16F10 cells, expression of these factors was measured using Western blot. Treatment with Erdr1 increased Bax expression, whereas Bcl-2 expression was decreased by Erdr1 treatment compared to control cells (Figure 3C). These results suggest that Erdr1 induces apoptosis in the murine melanoma cell line B16F10 via regulation of Bcl-2 and Bax.

Accumulating evidence indicates that inhibition of STAT3 activity results in the induction of apoptosis [11,12]. To determine the underlying mechanism of Erdr1-mediated apoptosis in melanoma, STAT3 activity following Erdr1 treatment was measured in B16F10 cells using DNA-binding ELISAs. The treatment of recombinant murine Erdr1 decreased the activity of STAT3 (Figure 3D). At 30 min after treatment of Erdr1, STAT3 activity was 22.3% ± 4.3% which is a significantly decreased level compared to the control (70.4% ± 7.0%). These results indicate that the Erdr1-induced apoptosis in melanoma cells is mediated by the inhibition of STAT3 activity.

## 3. Discussion

A defective apoptosis signaling pathway has been considered a main cause of chemoresistance in melanoma. Melanoma cell lines generally exhibit defects in the release of cytochrome c, which acts as a critical inducer of apoptosis [13]. Furthermore, high expression of Bcl-2 is correlated with resistance to chemotherapy in human melanomas and other tumors [14]. Therefore, identification of apoptosis regulators and the underlying mechanism will support advancements in melanoma therapy. In this study, we demonstrated that Erdr1 acts as an apoptosis-stimulating factor for melanoma via the downregulation of Bcl-2 and upregulation of Bax, ultimately leading to increased apoptosis in melanoma cells both *in vitro* and *in vivo*.

To identify the signaling molecules involved in Erdr1-mediated apoptosis, STAT3 activity was examined. Treatment of melanoma cells with Erdr1 inhibited STAT3 activity. STAT3 can be regulated by a variety of molecules including Janus kinase (JAK), mitogen activated protein kinase (MAPK) and Src. IL-6, which is involved in melanoma survival, can activate STAT3 through JAK [15]. Since STAT3 can be regulated by various molecules [16], further investigations are needed to determine whether STAT3 inhibition by Erdr1 is dependent on JAK or other upstream molecules of STAT3. In other studies, small molecule STAT3 inhibitors have been reported to induce apoptosis [12,17]. Furthermore, inhibition of STAT3 represses the expression of Bcl-2 and sensitizes cells to chemotherapy-induced apoptosis [18]. Various agents targeting anti-apoptotic Bcl-2 are currently in preclinical and clinical development because of their effective apoptosis induction [19]. In fact, ABT-737, a small molecule that targets anti-apoptotic Bcl-2 family proteins, has a synergistic effect in killing melanoma cells in combination with the proteasome inhibitor Bortezomib [20].

During melanoma progression, melanoma produces various aggressive characteristics which include chemoresistance, melanogenesis and metastasis. Melanogenesis is originally related to ultraviolet (UV) protection and can also affect melanoma behaviors. It has been known that melanin can attenuate chemotherapy and regulate immunosuppression [21]. Furthermore, according to Sarna *et al.* [22], melanin granules can impact the cell elasticity and inhibit transmigratory ability. Since Erdr1 is able to regulate not only apoptosis but also metastasis, how melanogenesis can be regulated by Erdr1 also needs to be further investigated.

In conclusion, this study revealed that Erdr1 suppresses tumor growth through the induction of apoptosis. Erdr1-mediated apoptosis was regulated by the downregulation of Bcl-2 and upregulation of Bax. Thus, Erdr1 acts as a novel regulator of apoptosis in melanoma.

## 4. Materials and Methods

### 4.1. In Vivo Tumorigenecity Model

Six-week-old C57BL/6 female mice (*N* = 8/group) were purchased from SLC Inc. (Haruno, Japan) and maintained for one week before experiments were begun. B16F10 cells (murine melanoma cell line) were purchased from ATCC. For examination of tumor growth, B16F10 cells (1 × 10^5^ cells/200 µL PBS) were subcutaneously injected into C57BL/6 mice. Thereafter, recombinant murine Erdr1 (100 µg/kg) or PBS were administered intraperitoneally every day for four weeks. Tumor size was determined by caliper measurement every two days. Four weeks after inoculation, mice were sacrificed, and tumor tissues were excised for further analysis of apoptosis and immunohistochemistry. Three independent experiments were performed. All animal experiments were approved by the Industry Academic Cooperation Foundation at Sookmyung Women’s University (Approval number: SMU-IACUC-2008-0902-001), and all experiments were conducted according to the regulatory standards.

### 4.2. Analysis of Apoptosis by Flow Cytometry

For detection of apoptotic cells in tumor tissues from mice using flow cytometry, cells were isolated from tumor tissues and stained with FITC conjugated Annexin V (Becton Dickinson, Franklin Lakes, NJ, USA) and 7-AAD (Biolegend, San Diego, CA, USA). Stained cells were analyzed using a fluorescence-activated cell sorting (FACS) Calibur (Becton Dickinson). To detect apoptotic cells in B16F10 cells following Erdr1 treatment, cells were treated with or without recombinant murine Erdr1 (1, 10, and 100 ng/mL) for 24 h and then stained with Annexin V and 7-AAD.

### 4.3. TUNNEL Assay

A TUNEL (terminal deoxynucleotidyl transferase-mediated d-UTP biotin nick end labeling) assay was performed using the TACS·XL-Blue label *in situ* apoptosis detection kit (Trevigen, Gaithersburg, MD, USA) according to the manufacturer’s protocol. Briefly, each section was rinsed with water and then equilibrated in PBS. The specimens were permeabilized, and the endogenous peroxidase activity was quenched by incubation in H_2_O_2_ in methanol. After rinsing, the sections were incubated with reaction buffer in a humidified chamber. Next, the reaction buffer was removed, and specimens were incubated with antibody solution. After subsequent incubation with a streptavidin-HRP solution, visualization of the reaction was performed using Trevigen Apoptotic Cell System (TACS) Blue Label solution. Nuclear Fast Red solution was used as a counter-stain.

### 4.4. Western Blot Analysis

Cells were washed twice with ice-cold PBS, and protein was extracted in ice-cold lysis buffer (50 mM Tris-HCl (pH 7.4), 1% NP-40, 0.25% deoxycholic acid sodium salt, 150 mM NaCl, 1 mM EDTA, and a protein inhibitor cocktail). After collecting the cell lysates, proteins were quantified using a Bradford assay (Bio-Rad, Hercules, CA, USA). An equal volume of protein from each sample was separated by 12% SDS-PAGE under reducing conditions and transferred to a polyvinylidene difluoride (PVDF) membrane (Bio-Rad). The membrane was blocked with a 5% non-fat milk solution for 1 h and then incubated with rabbit anti-mouse Bcl-2, Bax (Santa Cruz Biotechnology, Delaware, CA, USA), or α-tubulin antibodies (Sigma, Saint Louis, MO, USA) overnight. After washing, the membrane was incubated for 1 h with either goat anti-rabbit IgG antibody or goat anti-mouse IgG antibody conjugated with biotin followed by incubation with horseradish peroxidase for 30 min (Amersham Pharmacia Biotech, Buckinghamshire, UK). Each of the proteins was detected using an Amersham ECL system (Amersham Pharmacia Biotech).

### 4.5. Immunohistochemistry Analyses

Four weeks after tumor injection, tissue samples were embedded in optimal cutting temperature (OCT) compound and frozen. The samples were cut into 8 μm sections at −20 °C. Staining with hematoxylin and eosin (H & E) was performed for visualization of the nucleus and cytoplasm. To detect Bcl-2 and Bax expression in these tissue samples, immunohistochemistry was performed on the sections. Briefly, sections were fixed with cold acetone and then subjected to the blocking step with methanol containing 0.3% H_2_O_2_ for 30 min and 20% normal goat serum for 1 h. After blocking, sections were incubated with rabbit anti-mouse Bcl-2 or Bax antibody (1:100 dilution, Santa Cruz Biotechnology) for 1 h at room temperature. We subsequently added streptavidin-HRP (1:200 dilution, Molecular Probes, Carlsbad, CA, USA), and visualization was performed by adding 3,3’-diaminobenzidine (DAB). The sections were then counterstained with hematoxylin (Molecular Probes) for 5 min and then visualized under a microscope.

### 4.6. Cell Culture

B16F10 cells were cultured in DMEM supplemented with 2 mM l-glutamine, 100 units/mL penicillin, 100 μg/mL streptomycin, and 10% heat-inactivated fetal bovine serum. Cells were cultured at 37 °C in a humidified atmosphere containing 5% CO_2_. Cells in log phase were used for experiments.

### 4.7. Measurement of STAT3 Activity

TransAM^®^ STAT3 and STAT Family kits (Active Motif, Carlsbad, CA, USA) were used to detect STAT3 activity according to the manufacturer’s instruction. Briefly, cells were treated with or without recombinant Erdr1 (10 ng/mL) for 0, 15, 30, 60, and 120 min. Cell extracts were added into the oligonucleotide-coated wells. Subsequently, samples in each well were incubated with primary antibody and anti-IgG HRP conjugate for visualization. Processed STAT3 activity was analyzed using an ELISA microplate reader (Molecular Devices, Sunnyvale, CA, USA).

### 4.8. Statistics

Statistical analyses were performed with Prism 5 (GraphPad Software, La Jolla, CA, USA). The unpaired Student’s *t*-test was used and differences between the groups with *p*-values < 0.05 were considered statistically significant.
